# Rice (*Oryza sativa*) Stem Cells as a Novel Promising Active Ingredient with Anti-Proliferative Effects for Potential Skin Cancer Prevention and Skin Whitening Activity

**DOI:** 10.3390/foods13172803

**Published:** 2024-09-03

**Authors:** Leila Asadollahi, Soheil Abbaspour-Ravasjani, Kyung Ah Kim, Maryam Maghsoodi, Hamed Hamishehkar, Morteza Kosari-Nasab, Ki Hyun Kim

**Affiliations:** 1 Biotechnology Research Center, Student Research Committee, Faculty of Pharmacy, Tabriz University of Medical Sciences, Tabriz 51368, Iran; leilaasadollahi@tbzmed.ac.ir; 2 Drug Applied Research Center, Tabriz University of Medical Sciences, Tabriz 51368, Iran; abbaspour.s@tbzmed.ac.ir (S.A.-R.); kosarinasabm@gmail.com (M.K.-N.); 3 School of Pharmacy, Sungkyunkwan University, Suwon 16419, Republic of Korea; ruddk5480@naver.com; 4 Department of Pharmaceutics, Faculty of Pharmacy, Tabriz University of Medical Sciences, Tabriz 51368, Iran; mmaghsoodi@ymail.com; 5 Department of Plant, Cell and Molecular Biology, Faculty of Natural Sciences, University of Tabriz, Tabriz 51666, Iran

**Keywords:** rice callus, antioxidant activity, cytotoxicity, whitening, tyrosinase, melanoma inhibition, cosmeceuticals

## Abstract

Rice is one of the plants proven to possess antioxidant, anti-inflammatory, anti-proliferative, and whitening properties, making it one of the most beneficial plants in this regard. This study aimed to introduce a novel natural cosmetic and pharmaceutical product based on rice callus as a source of active ingredients that can inhibit skin melanoma cell (B16F10) proliferation and brighten the skin. The 2,4-D hormone at concentrations of 1 µg/mL and 1.5 µg/mL was used to induce rice callus formation. Rice callus extracts were then prepared using aqueous and ethanolic solvents, with a concentration of 1 mg/mL used for characterization tests. To determine the optimal hormone concentration, the phenols/flavonoids, antioxidant activity, proteins, and carbohydrates in the extracts were measured. The optimal concentration of the hormone was found to be 1 µg/mL. Finally, the anti-melanocyte and skin-whitening activity of the extracts was assessed through measurements of their cytotoxicity and inhibition of melanin synthesis-related enzymes. Cellular cytotoxicity measurements revealed that the ethanolic extract induced more cytotoxicity than the aqueous extract, with IC_50_ values of 566.3 µg/mL for the ethanolic extract and 1327 µg/mL for the aqueous extract. Skin-whitening-related tests demonstrated that the extracts were 1.7 times more effective than arbutin in inhibiting factors that cause hyperpigmentation. The aqueous extract achieved 85% inhibition of melanin biosynthesis at a concentration of 3200 µg/mL, compared to 68% for the ethanolic extract and 50% for arbutin. Based on these findings, rice callus extract can be introduced as a new, effective substance for skin-lightening and anti-melanocyte products in cosmeceutical and pharmaceutical formulations.

## 1. Introduction

The skin is a protective barrier and the most expansive organ protecting the body from harmful factors. Hyperpigmentation, a prevalent condition affecting all skin types, arises when there is excess production of melanin, the pigment responsible for skin color. This condition can be triggered by the increased activity of melanogenic enzymes, such as tyrosinase, or a higher number of melanocytes, the cells responsible for melanin synthesis [1]. Factors such as chronic inflammation, ultraviolet (UV) radiation, skin irritation, and abnormal α-melanocyte-stimulating hormone (α-MSH) release contribute to these disorders [2,3].

Skincare is one of the main factors that can protect against these skin-threatening elements. The use of plants has long been well known in this field due to certain compounds that improve the processes involved in the regeneration of skin cells. Phytochemicals, such as unsaturated fatty acids [4,5,6], carotenoids [5], saponins [7,8], phytohormones [9,10], vitamins [11], flavonoids [12], and alkaloids [13,14], have demonstrated significant benefits for skin health. Natural compounds such as polyphenols possess powerful antioxidant properties, which protect the skin from reactive oxygen species (ROS) and help to prevent skin aging [15]. The production of cosmeceuticals from plant resources is driven by secondary metabolites, and increasing the production efficiency of these compounds in the laboratory and their separation is one of the strongest biotechnological and even industrial and commercial attractions of these compounds. Plant-based natural products are being explored as novel forms of cosmeceuticals, particularly in skin whitening [16]. Many plant-derived ingredients, such as kojic acid and arbutin, have demonstrated positive effects on pigmentation reduction [17].

Skin cancer is among the most serious forms of cancer, primarily arising from uncorrected genetic mutations in skin cells’ DNA. These mutations can lead to abnormal cell growth and the development of malignant tumors [18]. Plant cell cultures have been utilized to manufacture anticancer substances, such as resveratrol sourced from *Arachis hypogea* callus cultures [19].

A new method involves using a sustainable and environmentally friendly source of raw materials: higher plants’ cell cultures and organs [20]. Plant cells can be grown in laboratories, creating a distinct biological system composed of a population of plant somatic cells. Initial research has shown significant differences between cells grown in vitro and those found in intact plants, particularly in terms of the cell growth rates and the synthesis and accumulation of bioactive compounds. These cultured cells exhibit vigorous division, with suspension cultures potentially producing more than a gram of dry biomass per liter of medium per day under optimal growth conditions [21]. Extracts obtained from calluses have been found to be more effective than those derived from various plant parts. In one study, a callus extract proved to be more effective than a leaf extract in reducing blood sugar levels in diabetic rabbits [22].

Rice is known for its well-documented properties, including anti-inflammatory, antioxidant, and skin-whitening effects [23]. In 1902, Austrian botanist Gottlieb Haberlandt discovered that adult plant cells could form calluses, which are masses of undifferentiated cells [24]. This foundational work laid the groundwork for plant tissue culture techniques, which were further validated in 1958 when a complete carrot plant was successfully regenerated from cultured in vitro carrot cells [25]. Since then, numerous articles have focused on plant regeneration from cultivated cells and tissue. The cosmetic industry, meanwhile, faces a significant challenge in delivering products that are not only functional and innovative but also trendy and safe for long-term use. Ethical concerns regarding the use of animal- or human-derived ingredients in cosmetics have further driven research and development toward biotechnology and plant cell culture technology. This shift aims to overcome industrial and regulatory constraints while meeting the consumer demand for sustainable and ethically produced products [26]. Stem cell extracts derived from plants have been reported to delay the aging process [27]. A callus, which is a mass of unusual and unknown cells that form in response to plant damage, is an example of this [28]. In simpler terms, a callus is a non-distinct, rapidly proliferating cell mass that can be produced by culturing small plant samples or seeds in nutrient media containing specific growth hormones [29]. Previous studies have highlighted the anti-aging properties of various plant stem cells, including those from grapes, tomatoes, raspberries, and medicinal plants [30]. 

The first documented case of the cytotoxic effects of a rice callus suspension culture was observed in colon and renal cancer cell lines, where it led to a significant reduction in cell viability of up to 95%. Interestingly, normal lung fibroblast cells remained unaffected at certain dilutions of the rice callus suspension culture. This cytotoxic effect was found to be as great as that of Taxol, a widely used anticancer drug, but without a harmful impact on healthy cells [31]. Moreover, the gas chromatography–mass spectrometry (GC-MS) analysis of yeast-fermented rice bran has revealed the presence of several metabolites, including phenolic compounds such as ferulic acid, p-coumaric acid, caffeic acid, and salicylic acid, as well as phytosterols like β-tocopherol and α-tocopherol. These compounds have been found to possess anti-inflammatory, antioxidant, and anti-proliferative properties [32].

Rice stem cells, however, have been studied only to a limited extent for their potential anti-aging and brightening effects [33,34,35]. Rice, scientifically known as *Oryza sativa*, contains a variety of phytochemicals, including γ-oryzanol, phenolic acids, and vitamin E homologs. These compounds are known to offer numerous health benefits, especially in the field of cosmetics [10,33]. The overproduction of melanin can lead to various undesirable effects, such as hyperpigmentation, post-inflammatory pigmentation, melasma, and premature skin aging. To achieve a lighter skin tone and counteract these effects, natural products like kojic acid and arbutin are well known for their skin-whitening and depigmenting properties. In addition to their depigmenting effects, these compounds have demonstrated anti-melanoma properties, including the ability to induce apoptosis in melanoma cells [36,37]. This study aims to investigate the novel potential of rice callus extract as an active ingredient for both skin cancer prevention and skin whitening. Specifically, it examines the extract’s anti-proliferative effects on melanoma cells and its ability to inhibit melanin synthesis. By evaluating these properties through standardized tests, the study seeks to establish rice callus extract as a promising novel ingredient in cosmeceuticals, enhancing skin health and combating skin discoloration, with benefits in both skin cancer prevention and skin lightening.

## 2. Materials and Methods

### 2.1. Materials

The Maman variety rice seeds used in this study were procured from the local market of Mianeh, located in Mianeh County, East Azerbaijan Province, Iran. The ethanol and sodium hypochlorite solutions were purchased from Dr. Mojallali Industrial Chemical Co., based in Tehran, Iran. Plant tissue culture media were obtained from HiMedia Laboratories LLC, headquartered in Maharashtra, India.

The following reagents and chemicals were also procured from Sigma Aldrich Co. in Hamburg, Germany: 2,4-D hormone, Folin–Ciocalteu reagent, aluminum chloride, sodium carbonate, gallic acid, potassium acetate, quercetin, 2,2-diphenyl-1-picrylhydrazyl (DPPH), sulfuric acid (98%), phenol, glucose, fructose, galactose, Bradford reagent, bovine serum albumin (BSA), SDS-PAGE materials, a protein ladder, RPMI 1640 medium, fetal bovine serum (FBS), cell culture antibiotics, trypsin–EDTA (0.25%), 4′,6-diamidino-2-phenylindole (DAPI), mono dansyl cadaverine (MDC), 2′-7′dichlorofluorescin diacetate (DCFH-DA), l-3,4-dihydroxyphenylalanine (l-DOPA), dimethyl sulfoxide (DMSO, 99.9%), phosphate-buffered saline (PBS), 3-(4,5-dimethylthiazol-2-yl)-2,5-diphenyltetrazolium bromide (MTT), and α-melanocyte-stimulating hormone.

### 2.2. Preparation of Callus and Extraction

To prepare the callus, the seeds were first immersed in a 70% (*v*/*v*) ethanol solution for 90 s and then thoroughly washed with a 20% sodium hypochlorite solution. The remaining sterilizing agents were removed by rinsing the grains four times with sterile water. The disinfected seeds were then grown aseptically in a supplemented MS basal medium containing different concentrations of the 2,4-D hormone. The culture containers were stored in the dark at 38 °C for 14 days [38]. After callus formation by the 2,4-D hormone, continuous callus growth, and multiple cultures, the desired cell line was selected based on morphological and biochemical characteristics. For extraction, 10 g of dried callus was carefully weighed. One group of calluses was extracted using 80% ethanol, while the other group was extracted using distilled water. The extraction was performed using an ultrasonic probe at room temperature, with 20 cycles of 1 min sonication followed by a 1 min rest, at a 40% amplitude (UP200H, Hielscher, Teltow, Germany).

### 2.3. Evaluating Total Phenols and Flavonoids of Callus Extracts

The Folin–Ciocalteu and aluminum chloride methods were used to determine the total concentrations of phenols and flavonoids, respectively [39,40]. Briefly, to evaluate the amount of total phenols, 100 µL of each callus extract was mixed with 400 µL of 75% sodium carbonate and 500 µL of the Folin–Ciocalteu reagent. Next, the prepared reaction mixture was incubated for 1 h at room temperature, and a UV–Vis spectrophotometer was used to assess the absorbance of the prepared mixture at 760 nm (Ultrospec 2000, Pharmacia Biotech, Cambridge, UK). The amount of total phenols was measured using the gallic acid standard solution calibration curve equation (mg GAE/g dry callus extract) [15,33]. In the subsequent phase of the experiment, the amount of total flavonoids in each extract was measured by mixing 500 µL of the extract with 100 µL of 10% (*w*/*v*) aluminum chloride solution and 100 µL of 0.1 mM potassium acetate solution. Then, the prepared mixture was diluted with 4.3 mL of distilled water and incubated at room temperature for 30 min. Finally, a UV–Vis spectrophotometer was used to assess the absorbance of this mixture at 415 nm. The amount of total flavonoids was calculated using the regression equation of quercetin standard solutions and characterized as quercetin equipollent (mg QCE/g dry callus extract).

### 2.4. Radical Scavenging Activity

The DPPH activity assay method was used to assess the activity of free radical scavenging. In this method, 100 µL of each sample was mixed with 900 µL of DPPH reagent and incubated in a dark place for 30 min. Then, using UV–Vis spectrophotometers, the absorbance was measured at 517 nm. The following equation was used to calculate the percentage of antioxidant activity [41]:

$$Antioxidant\;activity(\%)=\frac{\left(Absorbance\;of\;the\;control-Absorbance\;of\;the\;sample\right)}{Absorbance\;of\;the\;control}\times 100$$

### 2.5. Evaluating Total Carbohydrates

To measure the total carbohydrates, 500 µL of water extract was blended with 1 mL of phenol 2% and 2.5 mL of H_2_SO_4_ 98% and then kept in cool water for 40 min. A UV–Vis spectrophotometer was used to evaluate the absorbance of the mixture at 415 nm. The results were determined using stock solutions of glucose, fructose, and galactose as standards [42].

### 2.6. Measuring Total Protein

For the total protein assay of the water extracts, 50 µL of each sample was mixed with 2.5 mL Bradford reagent and shaken. The prepared mixture was incubated at room temperature for 5 min, and a UV–Vis spectrophotometer was used to measure the absorbance at 595 nm. The result was determined using a bovine serum albumin stock solution [43].

### 2.7. Investigation of Presence of Proteins

The presence of proteins in the water extracts was investigated via the SDS-PAGE technique. The top (4%) and bottom (12%) gels were prepared according to the protocol, and then the extracts were mixed with the sample buffer and added to the second well. In the first well, standard bands (Protein LADDER) were added. Then, the cassette was inserted into the tank, and the tank buffer was added. Afterward, the electrodes were connected to the device and set to a voltage of 200. After 35 min, the gel was removed from the machine and washed with the washing buffer three times for 30 s; it was then stained with Coomassie brilliant blue dye for 1 h. Finally, the gel was cleaned, and the molecular weight of the materials in the extracts was determined by the standard band map.

### 2.8. Cell Culture

To evaluate the melanocyte production inhibition and whitening properties of the obtained extracts, skin melanoma cancer cells (B16F10) were used (Pasteur Institute, Tehran, Iran). RPMI-1640 medium containing 10% FBS and 1% antibiotics was used as a cell culture medium. After three passages and the stabilization of the cells in the logarithmic stage, the cells were seeded at a density of 1.2 × 10^4^, 1 × 10^5^, and 2 × 10^5^ cells/well in 96-, 12-, and 6-well plates, respectively [44]. The treatment was initiated when the confluency reached 70% for the MTT assay, flow cytometry, and fluorescent imaging experiments. 

### 2.9. Detection of Cellular ROS

The DCFH-DA staining kit and flow cytometry analysis were used to detect and evaluate intracellular ROS accumulation. Briefly, 2 × 10^5^ cells per well were cultured in a six-well plate and incubated overnight. Next, the IC_50_ concentrations of the extracts determined from the DPPH assay results and by using vitamin C for 48 h before inducing oxidative stress with H_2_O_2_ were used to treat the cells. One hour after oxidative stress induction, the cells were subjected to 10 μL of DCFH-DA and incubated for two hours. Finally, the cells were detached using trypsin and rinsed twice with PBS. The intracellular ROS accumulation was evaluated by flow cytometry (FACSCalibur, BD, East Rutherford, NJ, USA) using an FL1-H band-pass filter (FITC) [44].

### 2.10. Cell Viability Study

To evaluate the cytotoxic effects of the extracts on B16F10 and 3T3 cells’ viability, the MTT assay was applied. After seeding the cells in a 96-well plate and overnight incubation, they were treated with different concentrations (0–4096 µg/mL) of 100 µL extracts. The media were replaced 48 h after treatment, and the cells were washed twice with PBS. Next, 150 µL of new medium containing 50 µL of MTT solution (2 mg/mL in PBS) was added to each well of the plate and incubated for 4 h. Then, the upper medium was substituted with 180 µL of DMSO containing 20 µL of Sorensen’s buffer and incubated for 30 min. Finally, the absorbance of the plates was read using an ELISA Reader (Sunrise™, TECAN, Männedorf, Switzerland) at a wavelength of 570 nm and a reference wavelength of 630 nm [45].

### 2.11. Apoptosis Assay

The apoptotic pathway of the cells was evaluated using flow cytometry. First, the cells were seeded in a 6-well plate and incubated overnight. Then, the upper culture medium was replaced with 2 mL of new media containing the IC_50_ concentration of each treatment, and the cells were incubated for 48 h. Finally, the cells were detached and stained with an annexin-V/PI apoptosis diagnostic kit (Exbio, Vestec-Jesenice u Prahy, Czech Republic), according to the manufacturer’s protocol. The apoptosis rate was evaluated via flow cytometry.

### 2.12. DAPI Staining

For the evaluation of DNA fragmentation in B16F10 cells, DAPI staining was performed. For this purpose, the cells were seeded on 12 mm cover glasses at the seeding compactness of 2 × 10^5^ cells/well. After 48 h of incubation, the cultured cells were treated with the IC_50_ concentrations of the treatments, and, after 48 h, they were washed with PBS twice and fixed with 4% formalin solution for 4 h. Next, the cells were washed again with PBS and incubated for 15 min in Triton X100 solution (0.01% *v*/*v*); after 15 min, the cells were exposed to DAPI solution (1 µg/mL) for 15 min. Finally, using a fluorescence microscopy system (BX50, Olympus, Tokyo, Japan), stained cells were observed to identify the apoptotic nuclei [46].

### 2.13. Discovery of Autophagic Vacuoles

Monodansylcadaverine (MDC) is an autofluorescent compound that accumulates in acidic autophagic vacuoles and is used to visualize and identify the process of autophagy and detect the level of autophagy pathway activation in B16F10 cells [47]. For this purpose, cells were seeded on 12 mm coverslips at a density of 2 × 10^5^ cells/well. After 48 h of incubation, the cultured cells were treated with the IC_50_ concentrations of the samples. Forty-eight hours after treatment, the cells were washed twice with PBS and stained with 0.05 mM MDC for 10 min at 37 °C. Subsequently, the cells were washed three times with PBS to remove excess MDC and imaged using a fluorescence microscope.

### 2.14. Prevention of Levodopa Oxidation

To evaluate the effects of rice callus extracts on the inhibition of tyrosinase activity, first, 100 µL of the sample solution (500–1400 µg/mL rice callus extract and arbutin as a control group in 0.1 M phosphate-buffered saline (PBS, pH 6.8)) was blended with 290 µL of PBS (0.1 M, pH 6.8) and 100 µL of 10 mM levodopa. Subsequently, the mixture was incubated for 5 min at 25 °C. Next, to the prepared cocktail, 10 µL of a mushroom tyrosinase solution (2500 units/mL in PBS (pH 6.8)) was added and it was incubated for 10 min at 37 °C once again [48]. Eventually, the absorbance was assessed at 475 nm, and the prevention of levodopa oxidation was calculated by the following equation:

Inhibition (%) = 100 − (B/A × 100)

A is the absorbance of the control at 475 nm, and B is the absorbance at 475 nm of the test sample.

### 2.15. Melanin Biosynthesis Inhibition in Melanocytes

Firstly, B16F10 melanoma cells (2 × 10^4^ cells/well) were sown in a 24-well plate and stimulated by 100 nM α-melanocyte-stimulating hormone (α-MSH) for 24 h to discover the preventive effects of the callus extracts on the biosynthesis of melanin in melanocytes. Then, the cells were treated with 50–3200 µg/mL (final concentration) of the callus extract or arbutin in 100 µL of RPMI medium. Next, the cells were incubated for 24 h and then washed two times with PBS (pH = 7.4), detached via trypsin, and collected as cell pellets. Afterward, the cell pellets were lysed with 150 µL of 1 N sodium hydroxide for 45 min at 65 °C. Eventually, the supernatants were collected, and the levels of melanin were assessed by reading the absorbance at 405 nm. The percentage of melanin synthesis inhibition was computed by comparing the levels of melanin in treated cells against those in untreated cells (100%) [48].

### 2.16. Western Blot Analysis

The total protein levels were extracted from the cells using the RIPA protein extraction kit (Santa Cruz Biotechnology, Dallas, TX, USA) and quantified using Nano-Drop spectroscopy. Before loading the proteins into the wells, they were mixed with sample buffer and boiled at 100 °C for 5 min. Next, 50 µg of protein per lane of whole cell lysate was electrophoresed in SDS-PAGE and transferred to a nitrocellulose membrane (Millipore, Burlington, MA, USA). Non-specific antigens were blocked using 2% non-fat milk, followed by incubation with specific primary antibodies (1:1000, Santa Cruz Biotechnology) at 4 °C for 18 h. The membrane was then washed three times with TBS-T buffer for 15 min each time, incubated with anti-rabbit secondary antibody (1:1000, Santa Cruz Biotechnology) for 75 min at room temperature, and subjected to chemiluminescence analysis [44]. The species and catalog numbers of the antibodies used in the Western blot experiments were as follows: β-Actin (C4): sc-47778, ERK 1/2 (H-72): sc-292838, p-ERK 1/2 (Thr 177)-R: sc-16981-R, Pan-Akt Polyclonal Antibody: Catalog No. E-AB-30471, Isotype IgG, p-Akt1/2/3 (B-5): sc-271966, Tyrosinase (T311): sc-20035.

### 2.17. Statistical Analysis

All experiments were repeated three times. Two-way ANOVA and independent *t*-tests with Tukey’s honest significant difference test for multiple comparisons were used for statistical analysis, performed using GraphPad Prism version 9.0.2 (San Diego, CA, USA). A *p*-value less than 0.05 was considered statistically significant. Data obtained from flow cytometry were analyzed using the FlowJo software (V10.5.3, Treestar Inc., San Carlos, CA, USA).

## 3. Results

### 3.1. Preparation and Characterization of Rice Callus Extract

In this study, the 2,4-D hormone at two different concentrations (1 and 1.5 µg/mL) was used to induce rice callus formation on agar-solidified basal MS medium complemented by gellan gum (0.7%) and sucrose (3%). After culturing the rice seeds, their calluses emerged with a dark yellow, compacted appearance. After collecting a sufficient amount of callus, they were dried in a dark place, and each gram was extracted with 10 mL of 80% ethanol and distilled water via a bath sonicator. Finally, to find the optimal concentration of the 2,4-D hormone, the prepared callus extracts were characterized, and the optimal concentration of the hormone was selected for further investigation based on these results.

**Figure 1:**
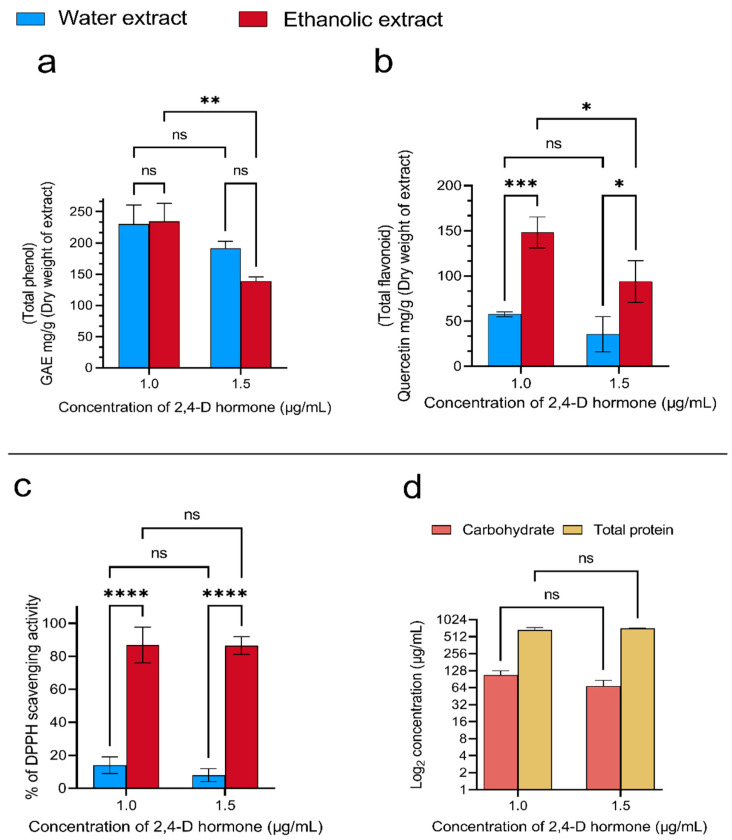
Rice callus extracts were analyzed for (**a**) total phenolic content, (**b**) flavonoid compounds, (**c**) 1,1-diphenyl-2-picrylhydrazyl (DPPH) free radical scavenging ability, and (**d**) total proteins and carbohydrates. Data are presented as mean ± standard deviation from three independent experiments. Statistical significance was determined using a one-way ANOVA with Tukey’s post hoc test (* *p* < 0.1, ** *p* < 0.01, *** *p* < 0.001, and **** *p* < 0.0001, ns = not significant).

The phenol/flavonoid content measurement results showed that increasing the concentration of the 2,4-D hormone in the culture media decreases the phenol/flavonoid concentration in the callus tissue (Figure 1a,b). Looking closer at the obtained numbers, increasing the hormone concentration from 1 ppm to 1.5 ppm reduces the amounts of the phenolic compounds in the aqueous and ethanolic extracts by 16% and 40%, respectively (Figure 1a). On the other hand, the results showed that the negative changes in the flavonoid concentrations were 38% and 37% in aqueous and ethanolic extracts, respectively (Figure 1b). 

**Figure 2:**
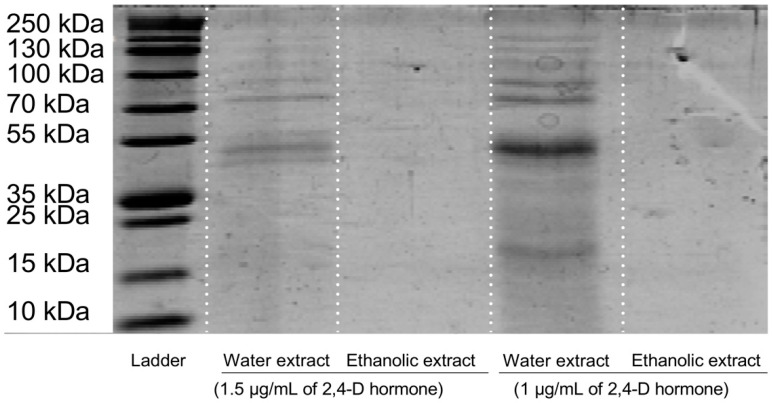
Protein macromolecule bands at two different concentrations of 2,4-D hormone treatment. The dotted lines were added to delineate the different extracts within the gel.

In the next stage of characterization, the antioxidant activity of the extracts was assessed by measuring their ability to inhibit free radicals using the DPPH reagent. The results showed that increasing the hormone concentration in the culture media of the water extracts led to the same results as described above. These results showed 43% inefficient free radical inhibition; however, there was no significant difference in the results for the ethanolic extracts (Figure 1c). In the final stage of the characterization, the carbohydrate and protein concentrations in the aqueous extract were measured (Figure 1d). The findings showed that increasing the hormone concentration led to the same results as described in the other characterization tests. The numbers showed a 36% reduction in the carbohydrate concentration, and there was no significant difference in the protein concentration when increasing the hormone concentration (Figure 1d). On the other hand, the ethanolic extract did not show any protein bands in the SDS-PAGE analysis. Still, for the water extracts, some bands appeared with a molecular weight of 15~25 kDa, 35~55 kDa, and 70~250 kDa (Figure 2). In conclusion, based on the characterization findings, the extracts of the callus obtained by one ppm hormone treatment were selected for further anti-melanoma and skin-lightening investigations.

### 3.2. Antioxidant Capacity Studies

**Figure 3:**
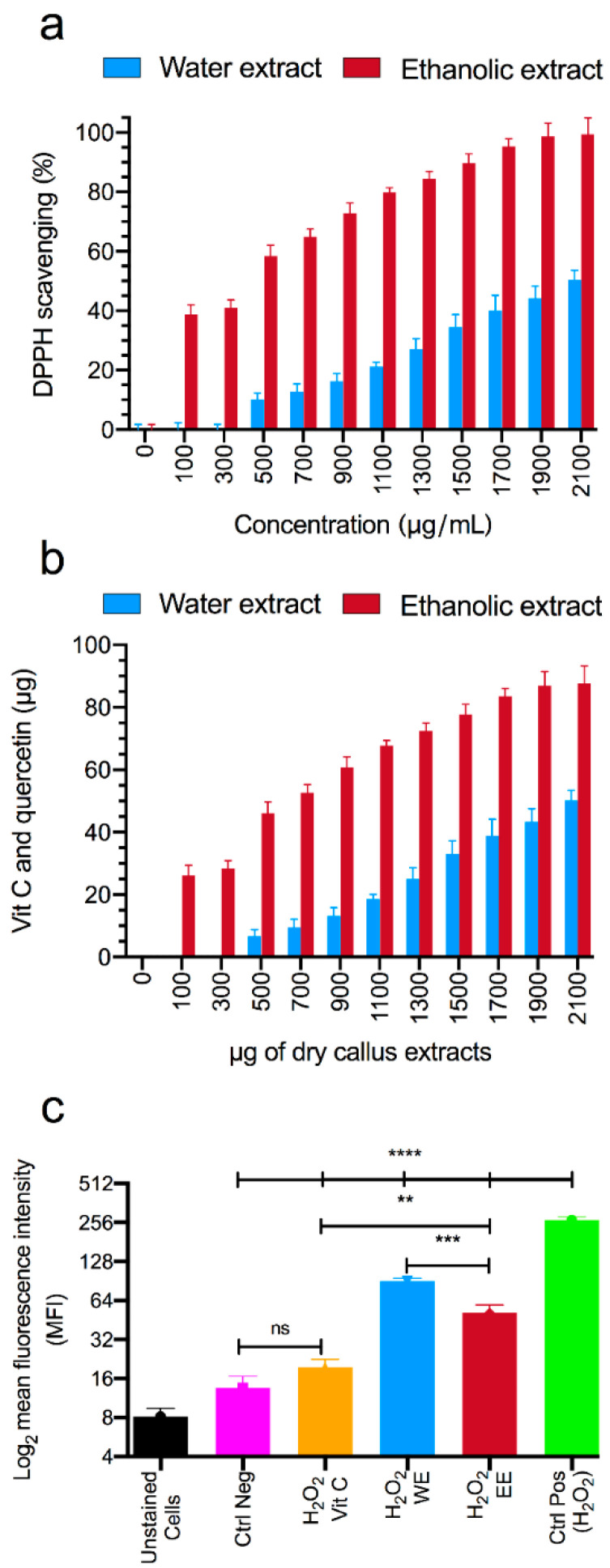
The ability of the extracts to inhibit DPPH free radicals (**a**). The antioxidant inhibition percentages of aqueous and ethanolic rice callus extracts standardized based on vitamin C and quercetin standards, respectively (**b**). The ability of the extracts to inhibit reactive oxygen species in murine melanoma (B16F10) cells (**c**). H_2_O_2_ was used as a positive control, while vitamin C and untreated cells served as negative controls. Data are presented as mean ± standard deviation from three independent experiments. Statistical significance was determined using a one-way ANOVA with Tukey’s post hoc test (** *p* < 0.01, *** *p* < 0.001, **** *p* < 0.0001, and ns = not significant).

After selecting the optimal concentration for callus growth, which was 1 μg/mL of the 2,4-D hormone, the antioxidant and free radical inhibition abilities of its ethanolic and aqueous extracts were measured. Figure 3a illustrates that the concentrations of 2152.86 μg/mL and 534.86 μg/mL, for the aqueous and ethanolic extracts, respectively, were able to inhibit 50% of the DPPH free radicals. Furthermore, by comparing the ethanolic extract with quercetin and the aqueous extract with vitamin C, we can conclude that each milligram of the ethanolic and aqueous extract possesses the same antioxidant capacity as 60 μg of quercetin and 15 μg of vitamin C, respectively (Figure 3b).

Finally, the ability of the ethanolic and aqueous extracts to reduce the intracellular ROS intensity using DCFH-DA fluorescent staining and flow cytometry equipment was evaluated. As shown in Figure 3c, the mean fluorescent intensity (MFI) of the cells increased from 13.54 to 267.35 after exposure to H_2_O_2_, without any additional treatment. However, when the cells were exposed to H_2_O_2_ and treated with the DPPH assay IC_50_ concentrations of the ethanolic and aqueous extracts, their MFIs decreased to 51.47 and 90.45, respectively. The statistical analysis revealed significant differences in the MFI between the ethanolic and aqueous extracts (*p* < 0.001). Additionally, treating the cells with these extracts significantly reduced their MFIs compared to H_2_O_2_-exposed cells (*p* < 0.0001).

### 3.3. Studies of Cellular Cytotoxicity of Rice Callus Extracts

**Figure 4:**
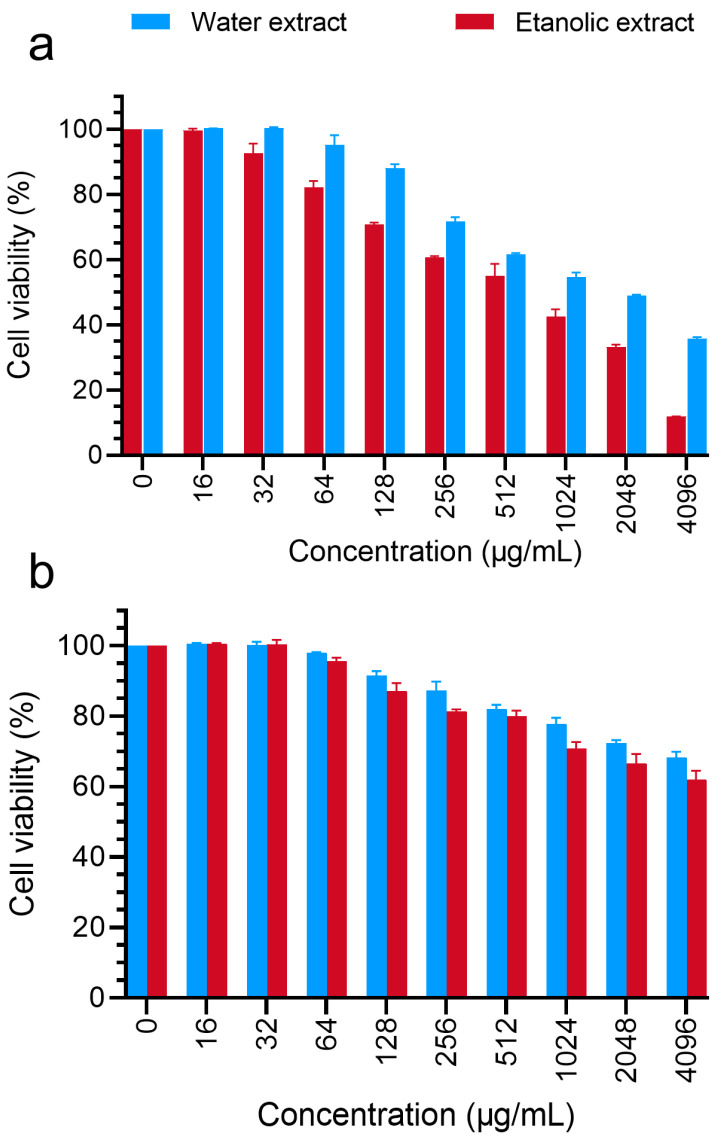
The cytotoxicity of the aqueous and ethanolic rice callus extracts was assessed in murine melanoma (B16F10) cells (**a**) and mouse embryonic normal fibroblast (3T3) cells (**b**). Data are presented as the mean ± standard deviation from three independent experiments.

To evaluate the anti-melanoma activity of the extracts, their cellular cytotoxicity activity against mouse melanoma cells (B16F10) was investigated using various cellular methods. In the first step, the cell cytotoxicity of the extracts against B16F10 and 3T3 cells was measured. The toxicity of the extracts on 3T3 cells was investigated to ensure little toxicity. The results showed a dose-dependent decrease in the viability of cells by the callus extracts after 48 h of treatment. After 48 h of treatment of B16F10 cells with the 4096 µg/mL concentration of the aqueous and ethanolic extracts, the viability of the cells was reported to be 35.8% and 11.9%, respectively, and the IC_50_ was reported to be 1327 µg/mL and 566.3 µg/mL, respectively (Figure 4a). According to the IC_50_ values, the toxicity of the ethanolic extract was 57% higher than that of the aqueous extract. Next, to prove the safety of the extracts against normal skin cells, their toxicity was measured against the 3T3 cell line. The findings showed that after 48 h of treatment, the viability of the cells at the 4096 µg/mL concentration of the aqueous and ethanolic extracts was 68.25% and 61.91%, respectively. Moreover, the IC_50_ values were reported to be 5744 μg/mL and 3957 μg/mL for the aqueous and ethanolic extracts, respectively (Figure 4b).

**Figure 5:**
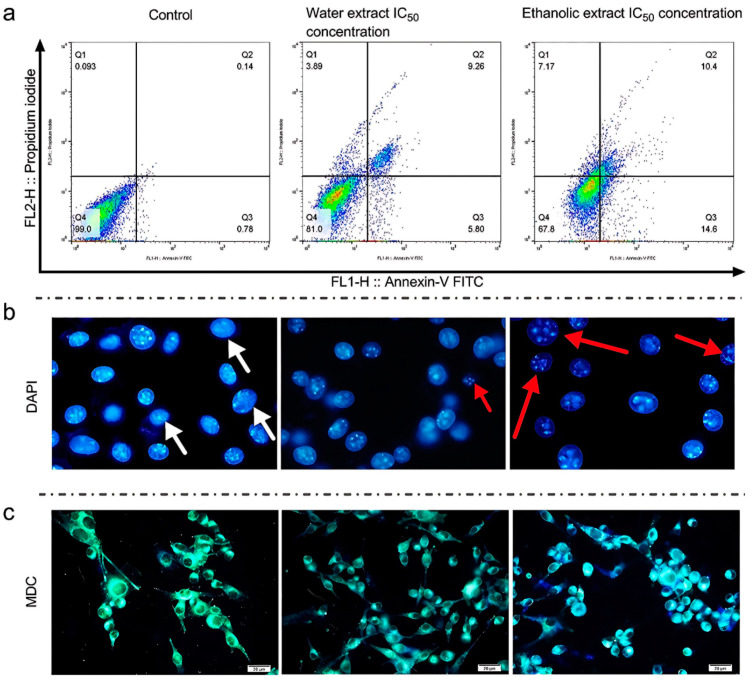
Evaluation of cell death pathways in murine melanoma (B16F10) cells treated with ethanolic and aqueous rice callus extracts. The apoptotic and necrotic effects were evaluated by annexin-V/propidium iodide (PI) staining methods (**a**), DNA fragmentation was visualized using 4′,6-diamidino-2-phenylindole (DAPI) staining (**b**), and autophagic vacuoles were detected using mono dansyl cadaverine (MDC) staining, with visible color shifts indicating autophagy activation (**c**).

In the next step of the cellular cytotoxicity study, the death pathways (apoptosis and necrosis) of the cells were investigated using an annexin V/PI kit. According to the results, the aqueous extract showed 3.89% necrosis and 15.06% apoptosis, while the ethanolic extract showed 7.17% necrosis and 25% apoptosis. The necrosis and apoptosis were reported to be 0.09% and 0.92% for the control group, respectively. The ethanolic extract was able to significantly increase the necrosis by 45.74% and apoptosis by 66%, compared to the aqueous extract (Figure 5a). The results of this test also reflected typical values for the control group. 

In the next step, the cell DNA was imaged to confirm the annexin V/PI results and visualize the DNA fragmentation. As shown in Figure 5b, the cells treated with the aqueous and ethanolic extracts demonstrated cellular DNA degradation. Furthermore, the investigation revealed that the amount of degraded DNA in the group of cells treated with the ethanolic extract was larger than in the aqueous extract (Figure 5b). 

In the final section of the cell death pathway evaluation, autophagy activation in B16F10 cells was assessed using MDC staining. According to Figure 5c, there were several autophagic vacuoles in the cells that were treated with both extracts. Looking closer at the images, an intense color shift from green to blue is visible. Moreover, the intensity of these color shifts as a sign of autophagy activation in the cells treated with the ethanolic extract was higher than in the cells treated with the aqueous extract.

### 3.4. Skin-Whitening Assays

**Figure 6:**
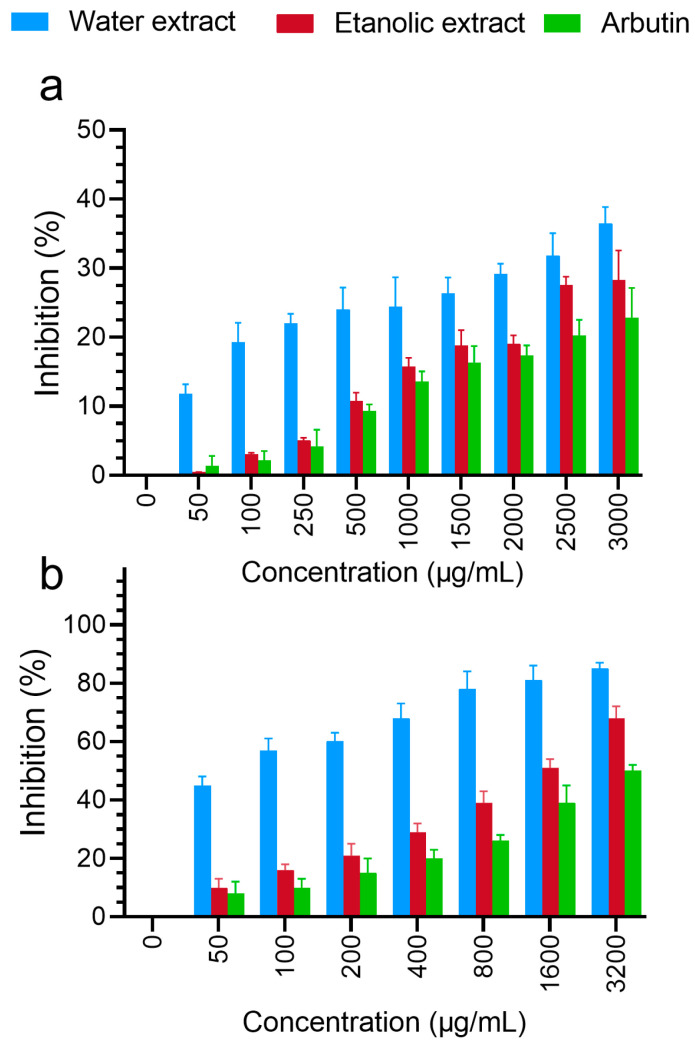
The ability of the rice callus aqueous and ethanolic extracts and arbutin to inhibit levodopa (L-DOPA) (**a**) and melanin biosynthesis (**b**) is shown. Data are presented as the mean ± standard deviation from three independent experiments.

The inhibition of levodopa oxidation by the aqueous and ethanolic extracts was measured. According to Figure 6a, the inhibition of levodopa oxidation increased with the increasing concentration in both groups. In general, the aqueous extract generally showed a higher rate of levodopa oxidation inhibition than the ethanolic extract. At the highest concentrations of the aqueous and ethanolic extracts (3000 µg/mL), the rate of levodopa oxidation inhibition was reported to be 36.44% and 28.30%, respectively (*p*-value = 0.0004). Moreover, the percentage of inhibition by arbutin as a control group at the same concentration was reported to be 22.78%, and, compared to the aqueous and ethanolic extracts, it decreased by 37.48% and 19.50%, respectively.

The inhibition of melanin biosynthesis by the aqueous and ethanolic extracts at different concentrations was measured and is demonstrated in Figure 6b. The inhibition of melanin biosynthesis increased with the increasing concentration in both groups. In general, the aqueous extract showed a higher percentage of inhibition than the ethanolic extract and the control group (arbutin). This percentage was reported to be 85% at the highest concentration (3200 µg/mL) for the aqueous extract and 68% and 50% for the ethanolic extract and the control group, respectively. The results showed that the extracts exerted a more significant inhibitory effect on melanin synthesis in melanoma cells than arbutin at the same concentration, as shown in Figure 6a,b. The inhibition of melanin biosynthesis by the aqueous extract was 20% greater than that of the ethanolic extract at the maximum concentration. The percentage of melanin biosynthesis inhibition with treatment with the ethanolic and aqueous extracts increased by 26.47% and 41.17% compared to that with arbutin in the control group, respectively.

### 3.5. Western Blot Analysis

**Figure 7:**
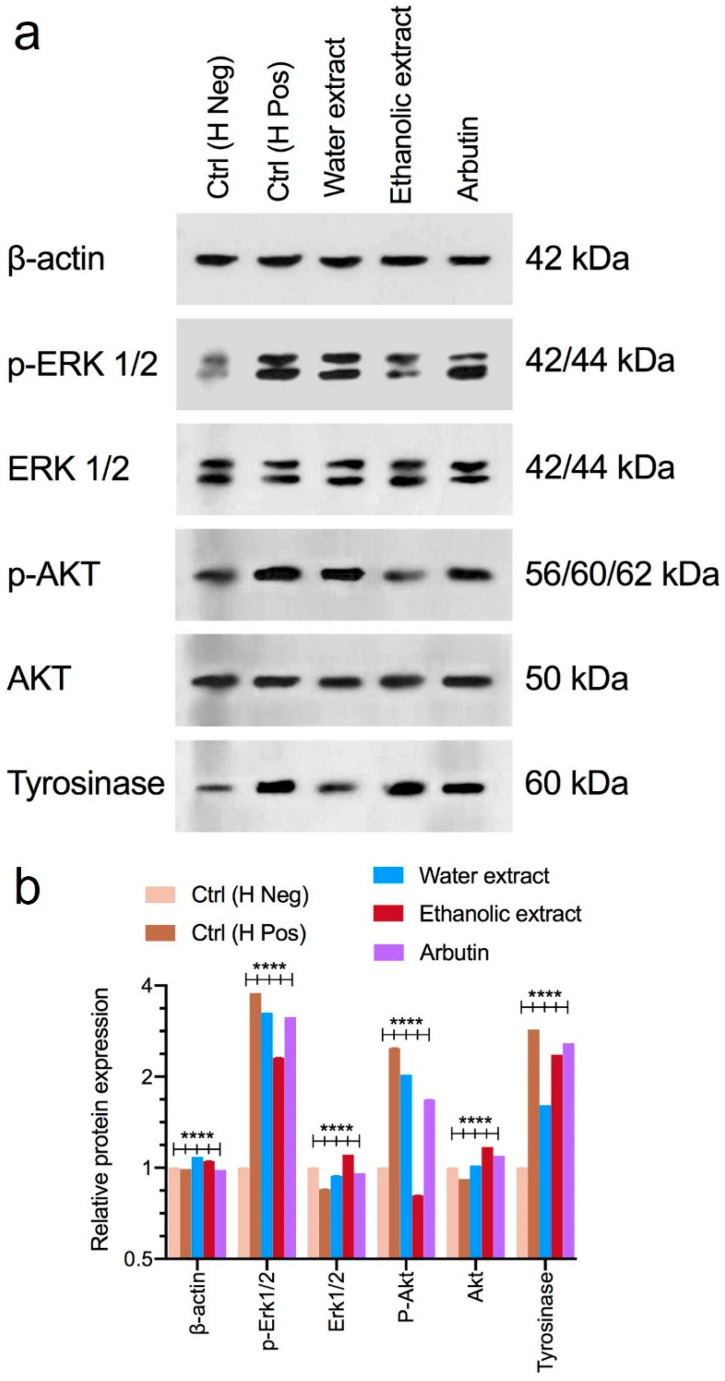
The protein levels of murine melanoma (B16F10) cells treated with rice callus ethanolic and aqueous extracts and arbutin are shown. Western blotting bands (**a**) and the expression intensity chart of the Western blotting bands (**b**). The α-melanocyte-stimulating hormone (α-MSH) without additional treatment was used as a positive control, while α-MSH-untreated cells served as negative controls. Data are presented as the mean ± standard deviation from three independent experiments. Statistical significance was determined using a one-way ANOVA with Tukey’s post hoc test (**** *p* < 0.0001 versus multiple comparisons).

The results of Western blotting, used to evaluate the expression of target proteins in B16F10 cells treated with the aqueous and ethanolic extracts, are shown in Figure 7. As shown in Figure 7, the results regarding the protein expression of ERK, p-ERK, AKT, and p-AKT (proteins involved in cell proliferation and melanogenesis) showed that the treatment of cells with α-MSH (positive hormone control) led to a significant increase in the p-ERK and p-AKT protein levels. When treating cells with the aqueous and ethanolic extracts, the expression of the p-ERK and p-AKT proteins was significantly reduced. The performance of the ethanolic extract compared to the aqueous extract was considerably better in inhibiting the proliferation and survival pathways and inhibiting melanogenesis. Arbutin was used as a commercial sample control, and it was found that its performance was between that of the aqueous and ethanolic extracts. In the second part of the study, the results showed that the addition of α-MSH to the cell culture medium led to a significant increase in the tyrosinase expression levels of the B16F10 cells, but, upon adding the aqueous and ethanolic extracts to the cell culture medium, the tyrosinase protein expression level significantly decreased. The aqueous extract had better performance compared to the ethanolic extract and it was better than the control, arbutin.

## 4. Discussion

The skin, as the body’s largest organ, serves multiple critical functions, including protection against external elements, the retention of body fluids, and defense against harmful microbes. Its quality is integral to human aesthetics, and hyperpigmentation—a condition marked by darker spots due to increased melanin production or altered melanogenic enzyme activity—can significantly impact the skin’s appearance. This condition is influenced by various internal and external factors [1]. Plant calluses, particularly rice calluses, are known for their rich content of active ingredients and secondary metabolites, such as phenolic compounds and proteins, which are valuable in cosmetic formulations [49,50]. Rice stem cell extracts, derived from embryonic cells in rice seeds, are rich in vitamins and exhibit potent antioxidant properties [30]. In this study, a rice callus extract was used to investigate the protective effects of this substance against hyperpigmentation and skin cancer. The phenolic compounds in such extracts have demonstrated antioxidant properties that help to mitigate skin disorders and prevent aging [51]. 

UV radiation significantly contributes to skin dullness and blemishes by generating free radicals that damage DNA and cause hyperpigmentation. Antioxidants can neutralize UV-induced oxidative damage, potentially reducing hyperpigmentation [52]. Plant calluses are recognized for their high antioxidant content [53], and plant secondary metabolites are crucial in pharmaceuticals, cosmetics, and nutrition [54]. These metabolites’ synthesis and accumulation are influenced by various factors, including the temperature, light exposure, and nutrient availability [55]. Optimizing these conditions in the lab could enhance the yields and effectiveness of these compounds for specific applications in the pharmaceutical and cosmetic industries. Our results indicate that both aqueous and ethanolic extracts contain flavonoids and phenolic compounds, with ethanolic extracts having higher concentrations. This aligns with the fact that ethanol is more effective in extracting these polar compounds due to their solubility [56]. Previous studies have shown that rice callus extract contains a high level of metabolites compared to extracts from some other plants [57]. 

In this study, the SDS-PAGE method was used to investigate the presence of proteins in the prepared extracts and their weights. The results showed mainly high-molecular-weight protein bands in the aqueous extracts, as seen in Figure 2, with no bar in the ethanolic fractions of these extracts, which was consistent with previous studies [58]. Given that high-molecular-weight proteins are predominantly found in aqueous extracts, future applications may include developing nano-formulated extracts for cosmetic and pharmaceutical use. Notably, our results showed that the total protein content in rice callus extracts is substantially higher than in rice seeds, as previously reported [59].

UV-induced oxidative stress leads to melanin production as a protective response, which can result in premature aging and skin damage [60]. Neutralizing the destructive effects of oxidative stress caused by UV radiation can represent a practical step in preventing the formation of skin spots. As mentioned above, the rice callus is rich in polyphenols and flavonoids, and it is expected to have a strong ability to inhibit free radicals in skin cells [15,61]. The DPPH assay results demonstrated that the antioxidant activity of the extracts increased in a concentration-dependent manner. The antioxidant inhibition concentrations (IC_50_) when evaluating the oxidative stress neutralization capacity against H_2_O_2_-induced oxidative stress in the cells showed better oxidative stress inhibition activity in the ethanolic fragment of the extract. The polarity of the polyphenols and flavonoids can explain the better antioxidant activity of the ethanolic fraction.

Antioxidants also play a role in combating melanoma by inhibiting unchecked melanocyte growth [62]. In a previous study, the protective effect of gamma oryzanol against UV-induced skin cancer was evaluated [41]. The results showed that this compound could efficiently protect mice’s skin against UV radiation, and, in vitro, it could reduce the viability of B16F10 cells through the activation of multiple death pathways. According to our results, the rice callus extracts could significantly increase the apoptotic cell numbers and significantly boost the intensity of autophagy in B16F10 cells. In this regard, previous studies have shown that a rice bran extract had significant toxicity in the B16F10 cell line [63], and our results showed that the rice callus extract was more cytotoxic than the rice bran extract against melanoma cells. Moreover, we evaluated the toxicity of the extracts on normal fibroblast cells to ensure negligible toxicity; our results showed no significant variability as compared to the control group (*p*-value < 0.0001).

The results of the annexin V/PI apoptosis tests are demonstrated in Figure 5a, and the results of the tests for autophagic vacuoles on the rate of DNA degradation by DAPI staining and MDC staining in B16F10 cells treated with the ethanolic and aqueous extracts and the control group are shown in Figure 5b,c; they confirm the results of previous tests. The sum of these results indicates the lightening effects of the extracts and their toxicity towards melanoma cells. The mentioned changes in the rates of cell death pathways and the improvements in these pathways favor the lightening and anti-melanoma activity of the extracts, which can change the cell death pathways by penetrating the cells. The results of this test also presented average values for the control group. Regarding apoptosis and cytotoxicity in melanoma cells and melanogenesis-inhibitory products, in a similar study by Yi-Shyan Chen et al. on Hispolon as a melanogenesis inhibitor, the survival rate of treated melanoma cells was 40.8% and the rate of cell apoptosis was the highest at 30.4% [64]. Moreover, in a study conducted by Liyan Jiang et al. on arbutin and its acetylated form as a skin brightener, the rates of apoptosis at the highest concentration in treated melanoma cells were 19.67% and 25.02%, respectively [65], which were close to our results.

To assess tyrosinase inhibition, a key enzyme in melanogenesis, we measured the inhibition of levodopa oxidation by various concentrations of the aqueous and ethanolic extracts. According to previous studies, plants can inhibit tyrosinase activity and, thus, melanin synthesis due to their different antioxidant compounds [66]. Compared with arbutin as the control group, both the ethanolic and aqueous extracts showed the more significant inhibition of levodopa oxidation (*p*-value < 0.001). Moreover, the results showed that the extracts had more significant inhibitory effects on melanin synthesis in melanoma cells than arbutin at the same concentration. As a whole, when increasing the concentrations of the extracts, the percentage of inhibition of melanin synthesis in melanocytes also increased. In a similar study by Deepak Adhikari et al. on the callus of Citrus Junos, the inhibition of levodopa oxidation at 500 µg/mL was reported at 25.2%, and the inhibition of melanin biosynthesis by the callus extract at 1 mg/mL was reported at 59.3%. It was 1.85 times greater than that of arbutin at the same concentration [48].

Western blot analysis was used to assess the impact of the rice callus extracts on key signaling pathways involved in melanin synthesis and melanoma cell proliferation. We observed that stimulation with a melanin-promoting hormone significantly increased the expression of p-AKT and p-ERK, with the ethanolic extract showing a stronger effect in neutralization than the aqueous extract and the control (arbutin). This is consistent with the cytotoxicity results. Tyrosinase is key to melanin production, and its inhibition is vital in preventing hyperpigmentation. The aqueous extract inhibited tyrosinase more effectively than the ethanolic extract, with both surpassing arbutin. The extracts likely exert their effects through multiple pathways. The ethanolic extract appears to enhance ERK1/2 and AKT activation, promoting the degradation of MITF, a key regulator of melanogenesis, thus reducing tyrosinase expression. The aqueous extract may directly inhibit tyrosinase through additional mechanisms. These results align with a recent study on a resveratrol-enriched rice callus extract, which also showed the significant suppression of MITF and tyrosinase via similar pathways [67]. This suggests that rice callus extracts, particularly those rich in bioactive compounds, hold promise as natural agents for the management of hyperpigmentation. 

This study has some limitations. First, the production of calluses is time-consuming and could be a challenge for large-scale industrial and cosmeceutical applications. To address this, a continuous production process should be developed to ensure a steady supply of callus extracts. Additionally, the research was conducted using a single melanoma cell line, which may limit the generalizability of the findings. Future research should explore the broader applicability of rice callus extracts across different cell lines, optimize the formulation concentrations, and assess the long-term safety for both cosmetic and pharmaceutical uses. However, this research highlights the potential of rice callus extracts as a natural ingredient for skin-lightening and anti-melanoma products. This could lead to the development of innovative cosmeceutical and pharmaceutical formulations.

## 5. Conclusions

This study evaluated the potential of rice callus extract as a natural ingredient for skin-brightening and anti-melanoma applications. Callus growth was optimized using 1 µg/mL and 1.5 µg/mL concentrations of the 2,4-D hormone, resulting in the preparation of aqueous and ethanolic extracts, which were then evaluated for their bioactive properties. The study conducted a comprehensive assessment of the extracts, including the phenolic/flavonoid content, antioxidant activity, and cytotoxicity against melanoma cells. The ethanolic extract demonstrated superior cytotoxicity and skin-whitening efficacy compared to arbutin. The study also explored the molecular mechanisms underlying melanin synthesis inhibition. Both extracts exhibited strong tyrosinase inhibition, with the aqueous extract showing a higher inhibition rate regarding melanin biosynthesis compared to the ethanolic extract. Our results showed that the ethanolic extract had more phenol/flavonoid content than the aqueous extract. Moreover, the ethanolic fraction could induce apoptosis in melanoma cells through multiple pathways. Our findings also indicate that the aqueous extract outperformed the ethanolic extract in inhibiting tyrosinase in vitro, aligning with its greater inhibition of tyrosinase gene expression. It was found to be more effective in inhibiting tyrosinase expression both in vitro and in gene expression than the control consisting of arbutin. Additionally, the aqueous extract showed a higher inhibition rate regarding melanin biosynthesis and the oxidation of levodopa than the ethanolic extract, and both inhibited melanin biosynthesis and the oxidation of levodopa more significantly than arbutin in the control group. Finally, after examining the expression of target proteins after the stimulation of melanoma cells by α-melanocyte-stimulating hormone, it was concluded that the aqueous and ethanolic extracts altered the expression of genes involved in melanogenesis and cell proliferation (ERK, P-ERK, AKT, P-AKT), as well as enabling the inhibition of tyrosinase gene expression. In summary, the ethanolic extract, being superior to the aqueous extract and arbutin as a control, inhibited the proteins involved in melanogenesis and cell proliferation, which was in line with the cytotoxicity results. In conclusion, both aqueous and ethanolic rice callus extracts demonstrate significant potential as effective agents for skin brightening and melanoma cell inhibition, indicating their applicability in cosmetic and pharmaceutical formulations.

## Data Availability

The data presented in this study are available on request from the corresponding authors. The data are not publicly available due to privacy restrictions.
